# Genetic Polymorphisms of Glutathione-Related Enzymes (*GSTM1*, *GSTT1*, and *GSTP1*) and Schizophrenia Risk: A Meta-Analysis

**DOI:** 10.3390/ijms160819602

**Published:** 2015-08-19

**Authors:** Su Kang Kim, Sang Wook Kang, Joo-Ho Chung, Hae Jeong Park, Kyu Bong Cho, Min-Su Park

**Affiliations:** 1 Kohwang Medical Research Institute, School of Medicine, Kyung Hee University, Seoul 130-701, Korea; E-Mails: skkim7@khu.ac.kr (S.K.K.); ifthisplus88@hanmail.net (S.W.K.); jhchung@khu.ac.kr (J.-H.C.); hjpark17@gmail.com (H.J.P.); 2 Department of Biomedical Laboratory Science, College of Health Sciences, Shinhan University, Gyeonggi 480-701, Korea; E-Mail: kbcho@shinhan.ac.kr; 3 Department of Surgery, School of Medicine, Kyung Hee University, Seoul 130-702, Korea

**Keywords:** glutathione *S*-transferase enzymes, polymorphism, schizophrenia, meta-analysis

## Abstract

The association between polymorphisms of glutathione-related enzyme (GST) genes and the risk of schizophrenia has been investigated in many published studies. However, their results were inconclusive. Therefore, we performed a meta-analysis to explore the association between the *GSTM1*, *GSTT1*, and *GSTP1* polymorphisms and the risk of schizophrenia. Twelve case-control studies were included in this meta-analysis. The odds ratio (OR) and 95% confidence interval (95% CI) were used to investigate the strength of the association. Our meta-analysis results revealed that *GSTM1*, *GSTT1*, and *GSTP1* polymorphisms were not related to risk of schizophrenia (*p* > 0.05 in each model). Further analyses based on ethnicity, *GSTM* polymorphism showed weak association with schizophrenia in East Asian population (OR = 1.314, 95% CI = 1.025–1.684, *p* = 0.031). In conclusion, our meta-analysis indicated the *GSTM1* polymorphism may be the only genetic risk factor for schizophrenia in East Asian population. However, more meta-analysis with a larger sample size were needed to provide more precise evidence.

## 1. Introduction

Schizophrenia is a mental disorder that is characterized by abnormal social behavior and altered perception of reality. Several studies suggested that genetic background and environmental factors play an important role in the development of schizophrenia. Recently, there has also been increasing interest in the role of free oxidative stress in the pathogenesis of schizophrenia. Oxidative damage and neuronal inflammation can lead to the onset of schizophrenia [1,2,3,4,5].

Glutathione *S*-transferases (GSTs) are a family of eukaryotic and prokaryotic phase II metabolic isozymes. GSTs are one of the key enzymes that regulate the conversion of toxic compounds to hydrophilic metabolites for the purpose of detoxification [6,7]. In addition, they play an important role as neuro-protective antioxidants by protecting neural cells from oxidative stress [4,8,9]. GSTs are expressed in many tissues in the human body. Some studies showed that GST levels of the cerebrospinal fluid were decreased in patients with schizophrenia [10]. It suggested that GSTs may play a significant role in the development and progression of schizophrenia.

Human cytosolic GSTs belong to the alpha, zeta, theta, mu, pi, sigma, kappa, and omega classes and the main *GSTs* genes described as polymorphic in humans are glutathione *S*-transferase Mu1 (*GSTM1*), glutathione *S*-transferase T1 (*GSTT1*), and glutathione *S*-transferase P1 (*GSTP1*) [11,12,13,14]. The *GSTM1* gene is located at 1p13.3, and the most common polymorphism in *GSTM1* is a deletion of the whole *GSTM1* gene with a lack of enzyme activity. The *GSTT1* gene is located at 22q11.2, and a homozygous deletion in *GSTT1* has also been reported. The *GSTP1* gene is located at 11q13, and the functional polymorphism at codon 105 in exon 5 of *GSTP1* has been found [11,12,13,14]. These polymorphisms of *GSTM1*, *GSTT1*, and *GSTP1* have been investigated with various diseases including schizophrenia, hypertension, and capacity for oxidation and detoxification [15,16]. Previous studies showed that *GSTM1* or *GSTT1* deletion polymorphisms were contributed to the loss of GST enzyme activities, respectively. So, combination in these *GSTM1* and *GSTT1* polymorphisms have been also investigated in order to find relationship with various diseases.

Up to now, a number of studies have evaluated the association between polymorphisms of *GSTs* gene and risk of schizophrenia in diverse populations. However, these results from the published studies remain conflicting rather than conclusive.

Therefore, we performed a meta-analysis on all eligible case-control studies to clarify the association between the *GSTM1*, *GSTT1*, and *GSTP1* polymorphisms and risk of schizophrenia.

## 2. Results and Discussion

### 2.1. Study Characteristics

The meta-analysis of this study was included 12 case and control studies. The characteristics from selected studies in *GSTM1*, *GSTT1*, and *GSTP1* polymorphisms and schizophrenia are summarized in Table 1. The 12 articles included 2742 schizophrenia patients and 2762 control subjects. The number of analyzed articles for *GSTM1*, *GSTT1*, and *GSTP1* polymorphisms were nine, eight, and four articles, respectively [3,17,18,19,20,21,22,23,24,25,26,27].

### 2.2. Quantitative Synthesis

**Figure 1:**
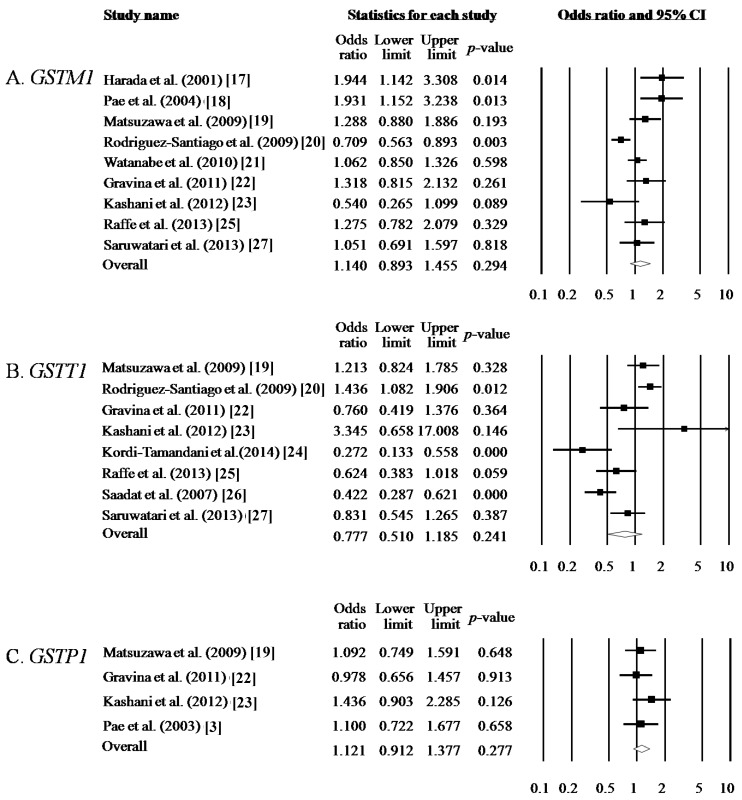
Odds ratio and 95% CIs are presented for individual studies (black square and line) and meta-analysis results (diamond). (**A**) Analysis of null type *vs*. present type of *GSTM1* polymorphism; (**B**) Analysis of null type *vs*. present type of *GSTT1* polymorphism; (**C**) Analysis of Val allele *vs*. Ile allele of *GSTP1* polymorphism.

The results of this heterogeneity test for meta-analysis are shown in Table 2. The random-effects method was applied if the result of the Q test was *p* < 0.05 or *I*^2^ statistic was >50%. Otherwise, the fixed-effects method was adopted. For *GSTM1* polymorphism, nine studies containing 2156 schizophrenia patients and 2289 control subjects were included. *GSMT1* polymorphism did not show any significant association with schizophrenia (null type *vs*. present type, OR = 1.140, 95% CI = 0.893–1.455, *p* = 0.294 in Table 2 and Figure 1A). For *GSTT1* polymorphism, eight studies containing 1703 schizophrenia patients and 1726 control subjects were included. *GSTT1* polymorphism did not show any significant association with schizophrenia (null type *vs*. present type, OR = 0.777, 95% CI = 0.510–1.185, *p* = 0.241 in Table 2 and Figure 1B). Combination of *GSTM1* and *GSTT1* polymorphisms were analyzed using meta-analysis. Combination of Null type in *GSTM1* and *GSTT1* polymorphisms were compared with combination of other types in *GSTM1* and *GSTT1* polymorphisms between the schizophrenia group and the control group. Combinations of *GSTM1* and *GSTT1* polymorphisms were not associated with schizophrenia (OR = 1.241, 95% CI = 0.866–1.777, *p* = 0.240 in Table 3).

For *GSTP1* polymorphism, four studies containing 659 schizophrenia patients and 562 control subjects were included. The genotype and allele of *GSTP1* were also not found to be associated with schizophrenia (codominant, dominant, recessive, and allele models, *p* > 0.05, respectively in Table 2 and Figure 1C).

When stratified for ethnicity of East Asian, *GSTM1* polymorphism showed weak association with schizophrenia (OR = 1.314, 95% CI = 1.025–1.684, *p* = 0.031). However, there was no association between, *GSTT1* or *GSTP1* polymorphisms and schizophrenia (*p* > 0.05 in Table 2 and Table 3).

To identify publication bias in meta-analysis, we evaluated publication bias using Egger’s regression. There was no publication bias (*p* > 0.05).

**Table 1:** Information of eligible studies included in the meta-analysis.

Authors	Population	Schizophrenia/Control	Schizophrenia							Control						
			*GSTM1*		*GSTT1*		*GSTP1*			*GSTM1*		*GSTT1*		*GSTP1*		
			Null	Present	Null	Present	Ile/Ile	Ile/Val	Val/Val	Null	Present	Null	Present	Ile/Ile	Ile/Val	Val/Val
Harada *et al.* (2001) [17]	Japanese	87/176	57	30	ND	ND	ND	ND	ND	87	89	ND	ND	ND	ND	ND
Pae *et al.* (2004) [18]	Korean	111/130	70	41	ND	ND	ND	ND	ND	61	69	ND	ND	ND	ND	ND
Matsuzawa *et al.* (2009) [19]	Japanese	214/220	129	85	88	127	154	55	5	119	101	80	140	162	54	4
Rodríguez-Santiago *et al.* (2009) [20]	Spanish	594/585	243	351	142	452	ND	ND	ND	289	296	105	480	ND	ND	ND
Watanabe *et al.* (2010) [21]	Japanese	627/620	336	291	ND	ND	ND	ND	ND	323	297	ND	ND	ND	ND	ND
Gravina *et al.* (2011) [22]	Italy	138/133	82	56	25	113	66	50	8	70	63	30	103	65	48	9
Kashani *et al.* (2012) [23]	Iran	93/99	15	78	6	87	48	37	8	26	73	2	97	64	27	8
Kordi-Tamandani *et al.* (2014) [24]	Iran	80/71	ND	ND	16	64	ND	ND	ND	ND	ND	34	37	ND	ND	ND
Pae *et al.* (2003) [3]	Korean	214/110	ND	ND	ND	ND	139	68	7	ND	ND	ND	ND	74	33	3
Raffa *et al.* (2013) [25]	Tunisia	138/123	79	59	59	79	ND	ND	ND	63	60	67	56	ND	ND	ND
Saadat *et al.* (2007) [26]	Iran	292/292	ND	ND	52	240	ND	ND	ND	ND	ND	99	193	ND	ND	ND
Saruwatari *et al.* (2013) [27]	Japanese	154/203	77	77	68	86	ND	ND	ND	99	104	99	104	ND	ND	ND
Total	-	2742/2762	1091	1065	456	1248	407	210	28	1136	1153	516	1210	365	162	24

ND, no data; *GSTM1*, glutathione *S*-transferase mu 1; *GSTT1*, glutathione *S*-transferase theta 1; *GSTP1*, glutathione *S*-transferase pi 1.

**Table 2:** Overall analysis between polymorphisms of glutathione *S*-transferase genes (*GSTM1*, *GSTT1*, and *GSTP1*) and susceptibility of schizophrenia.

Gene Symbols	Comparsions	Population	Heterogeneity		Model	OR	95% CI	*p*
			*p*	*I*^2^				
*GSTM1*	Null type *vs.* Present type	All	0.001	70.969	Random	1.140	0.893–1.455	0.294
		East Asian	0.090	50.291	Random	1.314	1.025–1.684	0.031
*GSTT1*	Null type *vs.* Present type	All	<0.001	83.745	Random	0.777	0.510–1.185	0.241
		East Asian	0.194	40.655	Fixed	1.020	0.767–1.355	0.893
*GSTP1*	Ile/Ile *vs.* Ile/Val	All	0.487	0.000	Fixed	1.167	0.905–1.504	0.235
	Ile/Ile *vs.* Val/Val	All	0.939	0.000	Fixed	1.144	0.640–2.043	0.650
	Ile/Ile *vs.* Ile/Val + Val/Val	All	0.533	0.000	Fixed	1.163	0.911–1.484	0.227
	Ile/Ile + Ile/Val *vs.* Val/Val	All	0.889	0.000	Fixed	0.771	0.435–1.368	0.374
	Ile *vs.* Val	All	0.666	0.000	Fixed	1.121	0.912–1.377	0.277
	Ile/Ile *vs.* Ile/Val	East Asian	0.945	0.000	Fixed	1.082	0.779–1.504	0.637
	Ile/Ile *vs.* Val/Val	East Asian	0.954	0.000	Fixed	1.279	0.490–3.339	0.615
	Ile/Ile *vs.* Ile/Val + Val/Val	East Asian	0.954	0.000	Fixed	1.907	0.797–1.510	0.569
	Ile/Ile + Ile/Val *vs.* Val/Val	East Asian	0.982	0.000	Fixed	0.956	0.367–2.490	0.927
	Ile *vs.* Val	East Asian	0.979	0.000	Fixed	1.095	0.827–1.450	0.525

OR, odds ratio; CI, confidence interval; *GSTM1*, glutathione *S*-transferase mu 1; *GSTT1*, glutathione *S*-transferase theta 1; *GSTP1*, glutathione *S*-transferase pi 1.

**Table 3:** Overall analysis between combination of glutathione *S*-transferase genes (*GSTM1* and *GSTT1*) and susceptibility of schizophrenia.

Gene symbols	Combination	Population	Heterogeneity		Model	OR	95% CI	*p*
			*p*	*I*^2^				
*GSTM1-GSTT1*	Null type *vs.* other types	All	0.958	<0.001	Fixed	1.241	0.866–1.777	0.240

OR, odds ratio; CI, confidence interval.

### 2.3. Discussion

Oxidative stress-mediated neuronal damage has been regarded as an important factor in the development of schizophrenia. We supposed that GSTs play an important role in antioxidant defenses by modulating various kinases, and we hypothesized that GSTs are related to the development of schizophrenia. [28,29,30]. In the present study, we examined the association between the polymorphisms of *GSTM1*, *GSTT1*, and *GSTP1* and the schizophrenia risk. To the best of our knowledge, this is the first meta-analysis to investigate the association between these polymorphisms and the development of schizophrenia.

The correlations between *GSTM1*, *GSTT1*, and *GSTP1* polymorphisms and the risk of schizophrenia have been investigated in a broad range of studies with either a relatively small or larger sample of the various populations. However, because of the differences in the number of participants and their genetic backgrounds, the evidence provided by each study is not sufficient to draw a convincing conclusion. We conducted a meta-analysis including with 2742 schizophrenia and 2762 controls from 12 case-control studies to evaluate the association between *GSTM1*, *GSTT1*, and *GSTP1* polymorphisms and the development of schizophrenia.

In our study, there were no significant associations between *GSTM1*, *GSTT1*, and *GSTP1* polymorphisms and risk of schizophrenia under any genetic model in the total population. Further subgroup analysis by ethnicity also demonstrated that only *GSTM1* polymorphism showed weak association with increased the risk of schizophrenia. We should also note the importance of heterogeneity. Heterogeneity was found in some comparisons in our meta-analysis. To get a more full and accurate detail of the precious data, we used the random-effects model. The results were stable with the sensitivity analysis, which did not change the results of the meta-analysis.

Some limitations remain in this meta-analysis and the results should be interpreted with caution. First, meta-analysis is a secondary and retrospective study type that is limited by the quality of primary studies. Second, we were unable to analyze gene-gene and gene-environment interactions.

This study investigated the relationship between *GSTM1*, *GSTT1*, and *GSTP1* polymorphisms and the development of schizophrenia. In conclusion, we found that *GSTM1* polymorphism was associated with susceptibility to schizophrenia in only East Asian population. Further larger studies considering gene-gene and gene-environment interactions are required to provide more precise evidence of the association between *GSTM1*, *GSTT1*, and *GSTP1* and the risk of schizophrenia.

## 3. Experimental Section

### 3.1. Search Strategy

Case and control studies were searched in PubMed, Google, Embase, and Korean databases (KISS, KMbase, and RISS) up to April 2015 without language restrictions. Relevant studies were identified using the terms: “glutathione-related enzyme” or “GSTM, or GSTT, or GSTP” and “polymorphism or polymorphisms” and “schizophrenia”. Only human studies were selected. Additional studies were identified by hand search of original or review articles. If data or data subsets were published in more than one article, only the publication with the largest sample size was included.

### 3.2. Inclusion Criteria

Studies were included if they met the following criteria: (1) they evaluated the association between the *GSTM1*, *GSTT1*, or *GSTP1* polymorphisms and schizophrenia; (2) they used a case-control study design; (3) and they contained sufficient distribution of *GSTM1*, *GSTT1*, or *GSTP1* polymorphism in the schizophrenia group and the control group for the estimation of an odds ratio (OR) with a 95% confidence interval (CI).

### 3.3. Data Extraction

The two investigators independently extracted data and formed an analysis. When they differed in their conclusions, they rechecked the data again and reached a consensus through discussion. Data extracted from the selected articles included the first author’s name, year of publication, country of origin, ethnicity of the study population, number of cases and controls, and the genotype frequency of *GSTM1*, *GSTT1*, or *GSTP1* polymorphisms.

### 3.4. Statistical Analysis

Meta-analysis was performed using a comprehensive meta-analysis software program (Corporation, NJ, USA). The pooled *p* value, OR and 95% CI were used to investigate the association between risk of schizophrenia and *GSTM1*, *GSTT1*, or *GSTP1* polymorphisms. Firstly, we calculated the heterogeneity of studies. A χ^2^-test-based *Q* statistic test and *I*^2^ test were applied. The random-effects DerSimonian and Laird method was adopted if the result of the *Q* test was *p* < 0.05 or *I*^2^ statistic was >50%, which indicated a statistically significant degree of heterogeneity between the studies. Otherwise, the fixed-effects Mantel-Haenszel method was adopted. Publication bias was evaluated by Egger’s regression.

For meta-analysis of *GSTM1* and *GSTT1* polymorphisms, the pooled ORs, 95% CI, and *p* value were calculated using null type versus (*vs*.) present type. And combination of *GSTM1* and *GSTT1* polymorphisms were also analyzed in meta-analysis [15]. For *GSTP1*, the pooled ORs were calculated for codominant 1 model (Ile/Ile *vs*. Ile/Val), codominant 2 model (Ile/Ile *vs*. Val/Val), dominant model (Ile/Ile *vs*. Ile/Val + Val/Val), recessive model (Ile/Ile + Ile/Val *vs*. Val/Val) and allelic model (Ile *vs.* Val). Further subgroup analysis by ethnicity, the East Asian population only included Koreans and Japanese. The value of *p* < 0.05 was regarded to indicate statistical significance.

## 4. Conclusions

*GSTM1* polymorphism was associated with susceptibility to schizophrenia in only East Asian population and *GSTT1* and *GSTP1* polymorphisms did not related to schizophrenia.
